# 5-(4-Methyl­phen­yl)-1,3,4-oxadiazol-2-amine

**DOI:** 10.1107/S1600536812019617

**Published:** 2012-05-12

**Authors:** Juan Zheng, Wen-juan Li, Manman Song, Yan Xu

**Affiliations:** a Department of Chemistry, Zhengzhou University, Zhengzhou 450052, People’s Republic of China

## Abstract

In the crystal structure of the title compound, C_9_H_9_N_3_O, adjacent mol­ecules are linked through N—H⋯N hydrogen bonds into a three-dimensional network.

## Related literature

For background to 1,3,4-oxadiazole derivatives, see: Lv *et al.* (2010 ▶); Bachwani & Sharma (2011 ▶); Padmavathi *et al.* (2009 ▶); Tang *et al.* (2007 ▶); Xue *et al.* (2007 ▶).
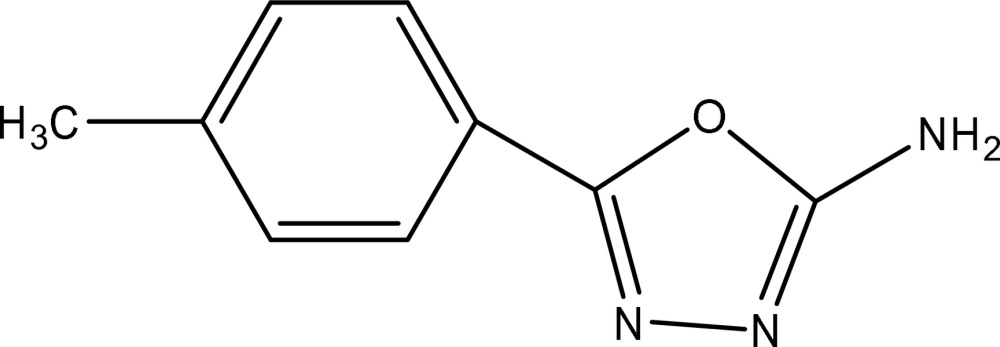


## Experimental

### 

#### Crystal data

C_9_H_9_N_3_O
*M* *_r_* = 175.19
Monoclinic, 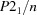

*a* = 12.161 (2) Å
*b* = 5.9374 (3) Å
*c* = 12.8282 (15) Å
β = 108.012 (19)°
*V* = 880.9 (2) Å^3^
*Z* = 4
Mo *K*α radiation
μ = 0.09 mm^−1^
*T* = 291 K
0.38 × 0.35 × 0.30 mm

#### Data collection

Rigaku Saturn diffractometer
Absorption correction: multi-scan (*CrystalClear*; Rigaku/MSC, 2006 ▶) *T*_min_ = 0.966, *T*_max_ = 0.973
3809 measured reflections
1800 independent reflections
1313 reflections with *I* > 2σ(*I*)
*R*_int_ = 0.022

#### Refinement

*R*[*F* ^2^ > 2σ(*F* ^2^)] = 0.045
*wR*(*F* ^2^) = 0.124
*S* = 1.03
1800 reflections
127 parameters
H atoms treated by a mixture of independent and constrained refinement
Δρ_max_ = 0.20 e Å^−3^
Δρ_min_ = −0.15 e Å^−3^

### 

Data collection: *CrystalClear* (Rigaku/MSC, 2006 ▶); cell refinement: *CrystalClear*; data reduction: *CrystalClear*; program(s) used to solve structure: *SHELXS97* (Sheldrick, 2008 ▶); program(s) used to refine structure: *SHELXL97* (Sheldrick, 2008 ▶); molecular graphics: *SHELXTL* (Sheldrick, 2008 ▶); software used to prepare material for publication: *SHELXTL*.

## Supplementary Material

Crystal structure: contains datablock(s) global, I. DOI: 10.1107/S1600536812019617/zj2066sup1.cif

Structure factors: contains datablock(s) I. DOI: 10.1107/S1600536812019617/zj2066Isup2.hkl

Supplementary material file. DOI: 10.1107/S1600536812019617/zj2066Isup3.cml

Additional supplementary materials:  crystallographic information; 3D view; checkCIF report

## Figures and Tables

**Table 1:** Hydrogen-bond geometry (Å, °)

*D*—H⋯*A*	*D*—H	H⋯*A*	*D*⋯*A*	*D*—H⋯*A*
N3—H3*A*⋯N1^i^	0.88 (2)	2.11 (2)	2.979 (2)	165.7 (19)
N3—H3*B*⋯N2^ii^	0.93 (2)	2.05 (2)	2.964 (2)	167.6 (16)

Symmetry codes: (i) 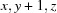; (ii) 
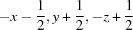
.

**Fig. 1.**
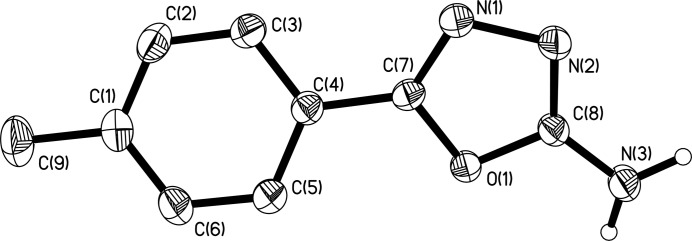
View of the title complex, showing the labeling of the 30% probability ellipsoids. H atoms are omitted for clarity.

**Fig. 2.**
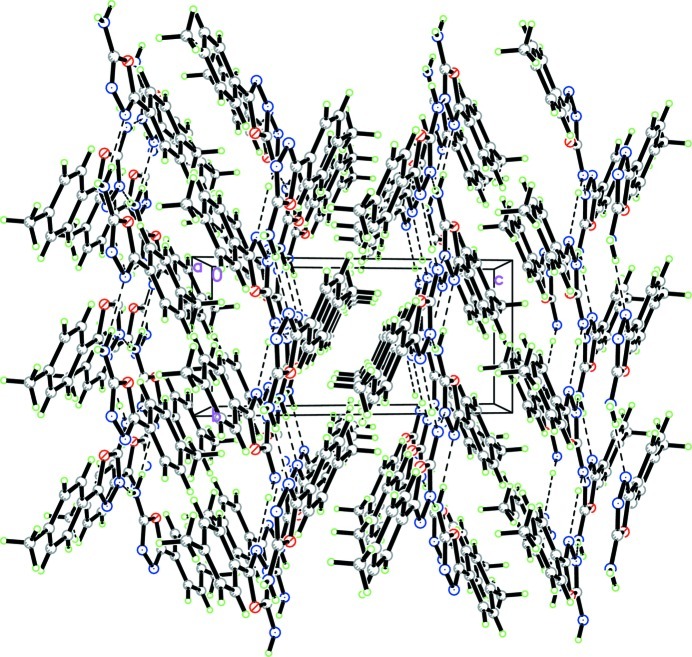
View of the title complex, showing the packing of the structure.

**Fig. 3.**
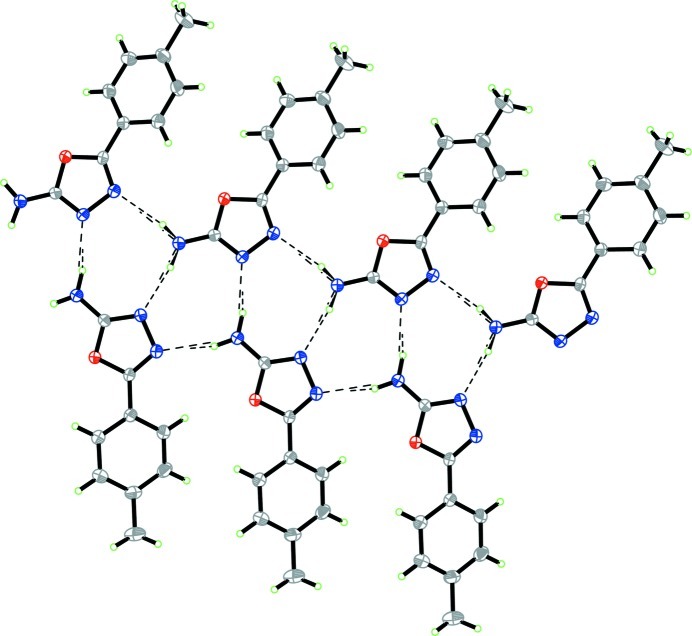
View of the title complex, showing the hydrogen bonding in the crystal structure.

## supplementary crystallographic information

### Comment

Oxadiazole is a five-membered heterocyclic aromatic chemical compound having two carbons, two nitrogen, and one oxygen atoms and two double bonds. Up to now, a large number of oxadiazole derivatives have been prepared and a series of novel substituted 1,3,4-oxadiazole derivatives were synthesized (Bachwani *et al.*, 2011). In addition, electron transporting 1,3,4-oxadiazole moiety has been connected to many chelating ligands to obtain luminescent complexes with more new function. (Lv *et al.*, 2010) 1,3,4-oxadiazole, which has abundant N-donor and O-donor sites is easily to form single-crystal. However, there has been limited study about their crystal properties. To further explore these types of structures, we synthesized the title compound and its crystal structure is presented herein. The molecular structure of the title compound is represented in Fig. 1. As shown in figure 1, the bond length between O1 with C8 is 1.3608 (19) Å and is nearly the bond length between O1 with C7(1.3754) Å. The angle of C8—O1—C7 is 102.79 (11) Å. Similarly, the bond length of C7 with N1 is approximate the bond length of C8 with N2. They are 1.279 (2) Å, 1.296 (2) Å. The bond length between N1 with N2 is 1.4129 (19) Å. The dihedral angle between the phenyl and the Oxadiazole ring bonded to the imino group is 26.37 °. The torsion angle between C(7)—N(1)—N(2)—C(8) is -0.3 (2) °. As depicted in figure 2 and 3, intramolecular N—H···N hydrogen bonds stabilize the molecular configuration.

### Experimental

The benzaldehyde (0.01 mol) and ethanol was added to semicarbazide hydrochloride (0.011 mol) refluxed 2 h. And then the obtained semicarbazone was oxidized by bromine liquid in acetic acid. The title compound (0.02 mmol) was dissolved in alcohol (3 ml) with a little aqueous solution. The resulting solution was allowed to stand at room temperature. Evaporation of the solvent, after three weeks yellow crystals with good quality were obtained from the filtrate and dried in air.

### Refinement

All H atoms are positioned geometrically and refined as riding atoms, with C—H = 0.93-0.98 Å, N—H = 0.86 Å, O—H = 0.82 Å, and with *U*_iso_ = 1.2*U*_eq_(C,N) or 1.5*U*_eq_(O).

### Crystal data

**Table:**

C_9_H_9_N_3_O	*F*(000) = 368
*M**_r_* = 175.19	*D*_x_ = 1.321 Mg m^−^^3^
Monoclinic, *P*2_1_/*n*	Mo *K*α radiation, λ = 0.71073 Å
*a* = 12.161 (2) Å	Cell parameters from 1122 reflections
*b* = 5.9374 (3) Å	θ = 3.3–26.3°
*c* = 12.8282 (15) Å	µ = 0.09 mm^−^^1^
β = 108.012 (19)°	*T* = 291 K
*V* = 880.9 (2) Å^3^	Prism, yellow
*Z* = 4	0.38 × 0.35 × 0.30 mm

### Data collection

**Table:**

Rigaku Saturn diffractometer	1800 independent reflections
Radiation source: fine-focus sealed tube	1313 reflections with *I* > 2σ(*I*)
Graphite monochromator	*R*_int_ = 0.022
Detector resolution: 28.5714 pixels mm^-1^	θ_max_ = 26.4°, θ_min_ = 3.3°
ω scans	*h* = −15→15
Absorption correction: multi-scan (*CrystalClear*; Rigaku/MSC, 2006)	*k* = −7→6
*T*_min_ = 0.966, *T*_max_ = 0.973	*l* = −16→15
3809 measured reflections	

### Refinement

**Table:**

Refinement on *F*^2^	Primary atom site location: structure-invariant direct methods
Least-squares matrix: full	Secondary atom site location: difference Fourier map
*R*[*F*^2^ > 2σ(*F*^2^)] = 0.045	Hydrogen site location: inferred from neighbouring sites
*wR*(*F*^2^) = 0.124	H atoms treated by a mixture of independent and constrained refinement
*S* = 1.03	*w* = 1/[σ^2^(*F*_o_^2^) + (0.0612*P*)^2^ + 0.0867*P*] where *P* = (*F*_o_^2^ + 2*F*_c_^2^)/3
1800 reflections	(Δ/σ)_max_ < 0.001
127 parameters	Δρ_max_ = 0.20 e Å^−^^3^
0 restraints	Δρ_min_ = −0.15 e Å^−^^3^

### Special details

**Table:**

Geometry. All e.s.d.'s (except the e.s.d. in the dihedral angle between two l.s. planes) are estimated using the full covariance matrix. The cell e.s.d.'s are taken into account individually in the estimation of e.s.d.'s in distances, angles and torsion angles; correlations between e.s.d.'s in cell parameters are only used when they are defined by crystal symmetry. An approximate (isotropic) treatment of cell e.s.d.'s is used for estimating e.s.d.'s involving l.s. planes.
Refinement. Refinement of *F*^2^ against ALL reflections. The weighted *R*-factor *wR* and goodness of fit *S* are based on *F*^2^, conventional *R*-factors *R* are based on *F*, with *F* set to zero for negative *F*^2^. The threshold expression of *F*^2^ > σ(*F*^2^) is used only for calculating *R*-factors(gt) *etc*. and is not relevant to the choice of reflections for refinement. *R*-factors based on *F*^2^ are statistically about twice as large as those based on *F*, and *R*- factors based on ALL data will be even larger.

### Fractional atomic coordinates and isotropic or equivalent isotropic displacement parameters (Å^2^)

**Table:**

	*x*	*y*	*z*	*U*_iso_*/*U*_eq_	
O1	0.00011 (9)	0.21279 (17)	0.19008 (10)	0.0417 (3)	
N1	−0.06440 (12)	−0.1327 (2)	0.19144 (13)	0.0516 (4)	
N2	−0.13933 (12)	0.0146 (2)	0.22353 (14)	0.0497 (4)	
N3	−0.13387 (15)	0.4123 (3)	0.24629 (15)	0.0572 (5)	
C1	0.29096 (15)	−0.2138 (3)	0.05483 (15)	0.0515 (5)	
C2	0.19911 (16)	−0.3566 (3)	0.04961 (15)	0.0537 (5)	
H2	0.1975	−0.4991	0.0191	0.064*	
C3	0.11020 (16)	−0.2923 (3)	0.08861 (15)	0.0485 (5)	
H3	0.0500	−0.3917	0.0847	0.058*	
C4	0.11017 (13)	−0.0803 (3)	0.13353 (14)	0.0395 (4)	
C5	0.20195 (15)	0.0649 (3)	0.14061 (16)	0.0497 (5)	
H5	0.2036	0.2073	0.1712	0.060*	
C6	0.29100 (15)	−0.0041 (3)	0.10172 (17)	0.0568 (5)	
H6	0.3524	0.0933	0.1073	0.068*	
C7	0.01433 (13)	−0.0122 (3)	0.17222 (14)	0.0388 (4)	
C8	−0.09732 (14)	0.2145 (3)	0.22099 (15)	0.0411 (4)	
C9	0.38709 (18)	−0.2859 (4)	0.0109 (2)	0.0749 (7)	
H9A	0.4075	−0.4395	0.0312	0.112*	
H9B	0.4533	−0.1910	0.0410	0.112*	
H9C	0.3617	−0.2730	−0.0676	0.112*	
H3A	−0.1032 (18)	0.535 (4)	0.2273 (17)	0.068 (6)*	
H3B	−0.2065 (17)	0.421 (3)	0.2559 (16)	0.062 (6)*	

### Atomic displacement parameters (Å^2^)

**Table:**

	*U*^11^	*U*^22^	*U*^33^	*U*^12^	*U*^13^	*U*^23^
O1	0.0408 (6)	0.0300 (6)	0.0602 (8)	−0.0006 (5)	0.0243 (6)	0.0001 (5)
N1	0.0544 (9)	0.0322 (8)	0.0790 (11)	−0.0021 (6)	0.0363 (8)	−0.0008 (7)
N2	0.0521 (8)	0.0319 (8)	0.0768 (11)	−0.0014 (6)	0.0372 (8)	0.0020 (7)
N3	0.0563 (10)	0.0337 (9)	0.0968 (14)	0.0003 (7)	0.0460 (10)	−0.0007 (8)
C1	0.0468 (10)	0.0584 (12)	0.0530 (11)	0.0107 (9)	0.0209 (9)	0.0035 (9)
C2	0.0652 (12)	0.0437 (10)	0.0584 (12)	0.0078 (9)	0.0279 (10)	−0.0056 (9)
C3	0.0538 (10)	0.0393 (9)	0.0573 (11)	−0.0033 (8)	0.0244 (9)	−0.0043 (8)
C4	0.0400 (8)	0.0351 (9)	0.0447 (10)	0.0027 (7)	0.0148 (7)	0.0025 (7)
C5	0.0478 (9)	0.0390 (10)	0.0654 (12)	−0.0004 (8)	0.0222 (9)	−0.0041 (9)
C6	0.0439 (10)	0.0566 (12)	0.0758 (14)	−0.0018 (9)	0.0269 (10)	0.0005 (10)
C7	0.0431 (9)	0.0279 (8)	0.0472 (10)	0.0011 (7)	0.0165 (8)	0.0016 (7)
C8	0.0394 (8)	0.0348 (9)	0.0544 (11)	−0.0004 (7)	0.0223 (8)	0.0020 (8)
C9	0.0596 (12)	0.0929 (17)	0.0811 (16)	0.0168 (11)	0.0350 (12)	−0.0082 (13)

### Geometric parameters (Å, º)

**Table:**

O1—C8	1.3608 (19)	C2—H2	0.9300
O1—C7	1.3754 (18)	C3—C4	1.385 (2)
N1—C7	1.279 (2)	C3—H3	0.9300
N1—N2	1.4129 (19)	C4—C5	1.391 (2)
N2—C8	1.296 (2)	C4—C7	1.458 (2)
N3—C8	1.331 (2)	C5—C6	1.387 (2)
N3—H3A	0.88 (2)	C5—H5	0.9300
N3—H3B	0.93 (2)	C6—H6	0.9300
C1—C6	1.382 (3)	C9—H9A	0.9600
C1—C2	1.388 (3)	C9—H9B	0.9600
C1—C9	1.509 (3)	C9—H9C	0.9600
C2—C3	1.378 (2)		
			
C8—O1—C7	102.79 (11)	C6—C5—C4	119.58 (16)
C7—N1—N2	107.39 (13)	C6—C5—H5	120.2
C8—N2—N1	105.34 (13)	C4—C5—H5	120.2
C8—N3—H3A	117.2 (13)	C1—C6—C5	121.82 (17)
C8—N3—H3B	119.0 (11)	C1—C6—H6	119.1
H3A—N3—H3B	119.1 (17)	C5—C6—H6	119.1
C6—C1—C2	117.61 (16)	N1—C7—O1	111.77 (14)
C6—C1—C9	121.48 (18)	N1—C7—C4	129.48 (15)
C2—C1—C9	120.92 (18)	O1—C7—C4	118.74 (13)
C3—C2—C1	121.55 (17)	N2—C8—N3	129.62 (16)
C3—C2—H2	119.2	N2—C8—O1	112.70 (14)
C1—C2—H2	119.2	N3—C8—O1	117.65 (14)
C2—C3—C4	120.28 (17)	C1—C9—H9A	109.5
C2—C3—H3	119.9	C1—C9—H9B	109.5
C4—C3—H3	119.9	H9A—C9—H9B	109.5
C3—C4—C5	119.15 (16)	C1—C9—H9C	109.5
C3—C4—C7	119.79 (15)	H9A—C9—H9C	109.5
C5—C4—C7	121.06 (15)	H9B—C9—H9C	109.5
			
C7—N1—N2—C8	−0.3 (2)	N2—N1—C7—C4	−177.86 (16)
C6—C1—C2—C3	0.6 (3)	C8—O1—C7—N1	−0.76 (19)
C9—C1—C2—C3	−179.45 (18)	C8—O1—C7—C4	177.94 (14)
C1—C2—C3—C4	0.6 (3)	C3—C4—C7—N1	14.9 (3)
C2—C3—C4—C5	−1.2 (3)	C5—C4—C7—N1	−165.33 (19)
C2—C3—C4—C7	178.60 (16)	C3—C4—C7—O1	−163.57 (15)
C3—C4—C5—C6	0.6 (3)	C5—C4—C7—O1	16.2 (2)
C7—C4—C5—C6	−179.16 (17)	N1—N2—C8—N3	−178.37 (19)
C2—C1—C6—C5	−1.2 (3)	N1—N2—C8—O1	−0.2 (2)
C9—C1—C6—C5	178.88 (19)	C7—O1—C8—N2	0.58 (19)
C4—C5—C6—C1	0.6 (3)	C7—O1—C8—N3	178.98 (16)
N2—N1—C7—O1	0.7 (2)		

### Hydrogen-bond geometry (Å, º)

**Table:**

*D*—H···*A*	*D*—H	H···*A*	*D*···*A*	*D*—H···*A*
N3—H3*A*···N1^i^	0.88 (2)	2.11 (2)	2.979 (2)	165.7 (19)
N3—H3*B*···N2^ii^	0.93 (2)	2.05 (2)	2.964 (2)	167.6 (16)

Symmetry codes: (i) *x*, *y*+1, *z*; (ii) −*x*−1/2, *y*+1/2, −*z*+1/2.
